# Feasibility of Discriminating UAV Propellers Noise from Distress Signals to Locate People in Enclosed Environments Using MEMS Microphone Arrays

**DOI:** 10.3390/s20030597

**Published:** 2020-01-21

**Authors:** Alberto Izquierdo, Lara del Val, Juan J. Villacorta, Weikun Zhen, Sebastian Scherer, Zheng Fang

**Affiliations:** 1 Signal Theory and Communications Department, University of Valladolid, 47011 Valladolid, Spain; juavil@tel.uva.es; 2 Mechanical Engineering Area, Industrial Engineering School, University of Valladolid, 47011 Valladolid, Spain; lvalpue@eii.uva.es; 3 Department of Mechanical Engineering, Carnegie Mellon University, Pittsburgh, PA 15289, USA; weikunz@andrew.cmu.edu (W.Z.); basti@andrew.cmu.edu (S.S.); 4 Faculty of Robot Science and Engineering, Northeastern University, Shenyang 110819, China; fangzheng@mail.neu.edu.cn

**Keywords:** unmanned aerial vehicle (UAV), acoustic array, people localization, environments with reduced visibility, enclosed environments

## Abstract

Detecting and finding people are complex tasks when visibility is reduced. This happens, for example, if a fire occurs. In these situations, heat sources and large amounts of smoke are generated. Under these circumstances, locating survivors using thermal or conventional cameras is not possible and it is necessary to use alternative techniques. The challenge of this work was to analyze if it is feasible the integration of an acoustic camera, developed at the University of Valladolid, on an unmanned aerial vehicle (UAV) to locate, by sound, people who are calling for help, in enclosed environments with reduced visibility. The acoustic array, based on MEMS (micro-electro-mechanical system) microphones, locates acoustic sources in space, and the UAV navigates autonomously by closed enclosures. This paper presents the first experimental results locating the angles of arrival of multiple sound sources, including the cries for help of a person, in an enclosed environment. The results are promising, as the system proves able to discriminate the noise generated by the propellers of the UAV, at the same time it identifies the angles of arrival of the direct sound signal and its first echoes reflected on the reflective surfaces.

## 1. Introduction

There are a large number of systems on the market to locate and track people. The solutions can be divided into two categories: one based on sensors that explore a surveillance space, and the other one based on devices that people carry on and that are detected by a control network [1,2].

Sensor-based solutions detect people without their active collaboration. Among these solutions, the following systems can be highlighted, those based on: (i) cameras, either in the visible and in the infrared (thermal image) frequency bands [3,4], (ii) cameras with structured light patterns, which measure the range of each pixel (RGB (red, green and blue) depth) [5], (iii) LIDAR (Light Detection and Ranging) systems [6,7], (iv) ultra-wide band RADARs (Radio Detection and Ranging) [8,9] and (v) acoustic systems (microphones, arrays of microphones, etc.) [10,11].

On the other hand, device-based solutions require that each person carries a device on, which can be identified through a monitoring network. The most common devices are RFID (radio frequency identification) Tags [12,13], GPS (global positioning system) Tracker [14,15], Cellular Phones [16,17,18], BLE (Bluetooth low energy) Beacons [19,20,21] and Wi-Fi devices (tablet, portable, cellular, etc.) [22].

It is difficult to select one system, among all these possibilities, to detect people in an enclosed space, where the following circumstances can take place:

1. There is no sensor network installed, or is not operational.
2. There is an emergency caused by a fire or an explosion with dense smoke.
3. Anonymous people to be located, not knowing their identity a priori.

If people who may be present are not known, it invalidates device-based solutions, since it is unknown who they are and whether they carry any generic or specific operational device. So, under these circumstances, sensor-based solutions would be the right choice to detect people using autonomous systems (robots, drones, etc.). These systems are usually based on: (i) image identification; (ii) analysis of signals disturbed by the movement of people; and (iii) detection of sounds generated by people (whistles, cries for help, etc.). In this way, these systems are not dependent on any previous sensor installation or the sensor operability.

Image-based sensors will not work in environments with reduced visibility, such as environments with dense smoke and severe fire. In these cases, it is necessary to rely on ultra-wide band radars or smart acoustic systems, which are not based on optical image. Radar-based systems are very effective even at detecting people behind walls, but have a small operational range and high cost and weight/energy consumption.

Smart acoustic systems are based on arrays, which are arranged sets of identical sensors, fed in a specific manner. The beampattern of an array can be controlled by modifying its geometry (linear, planar, etc.), the sensor spacing, and the amplitude and phase excitation of each sensor [23]. Microphone arrays are a particular case of these systems. By using beamforming techniques [24], the array beampattern, particularly its main lobe, can be electronically steered to different spatial positions, allowing spatial filtering, i.e., the discrimination of acoustic sources based on their position. These arrays are used in applications such as speech processing, echo cancellation, sound sources separation and localization [25].

To get 2D spatial information, working with sensors distributed on a surface (planar arrays) is necessary. Using planar arrays leads to a high system complexity and cost, where the acoustic sensors and the associated hardware require a large space. The acronym MEMS (micro-electro-mechanical system) refers to mechanical systems with a dimension smaller than 1 mm, which are manufactured with tools and technology arising from the integrated circuits (ICs) field [26]. The application of MEMS technology to acoustic sensors has allowed the development of high-quality microphones with high SNR (signal to noise ratio), low power consumption, and high sensitivity [27]. The combination of MEMS microphones with FPGA-based architectures are very common in applications based on sound sources localization [28]. An acquisition and processing system for an acoustic array, based on digital MEMS microphones, is reduced to two basic elements: MEMS microphones and a processing system. The integration of the microphone preamplifier and the ADC (analog to digital converter) in a single chip significantly reduces costs and the space occupied by the system. The most recent acoustic system developed by the authors from the University of Valladolid, and used in this work, is based on a planar array of digital MEMS microphones [29].

The Robotics Institute of the Carnegie Mellon University (CMU), managed by Professor Sebastian Scherer, has a working line that is based on flying unmanned aerial vehicles (UAV), or drones, inside visually degraded enclosed environments to locate people and fire focuses [30]. Inside these environments, navigating using GPS is not possible. This is the reason why the drone has to navigate autonomously, at the same time as it generates 3D maps, using SLAM (simultaneous localization and mapping) optical techniques [7,31,32,33,34]. This navigation system uses a FLIR (Forward-Looking Infrared) thermal camera to detect fire and an RGB depth camera to navigate [30].

Within this framework, that is, with an UAV flying in enclosed environments, where visibility could be reduced, these RGB cameras do not show a good behavior, and the performance of the thermal ones is very limited. So, under these circumstances, an idea arose to analyze if it would be feasible to include an acoustic array on a drone, to detect people in danger from their cries for help.

There are studies were the acoustic arrays are used to detect and track the position of drones [35,36]. The signals acquired by the microphones are processed to detect the noise produced by the drone propellers, which is strong enough to be eared form the distance. The noise level will increase, as the array is closer to the drone and may mask other acoustic sources. In this paper, based in this idea, the authors study the feasibility of discriminating UAV propellers noise from distress signals to locate people in danger, using an array of MEMS microphones.

Although this paper is based on discriminating distress signals from only propellers noise, this kind of noise is not the only noise source that could be present in real scenarios. Ambient and fire noises could be also present in a real scenario. These kinds of noises have a low frequency spectrum, and therefore, in analogy to the noise generated by the drone propellers, they could be discriminated using a high pass filter. In addition, the intensity level of these noise sources is significantly lower than the noise generated by the propellers that are at a very small distance from the array of microphones. Other possible noise sources could be related with explosions. This kind of noises would have an intensity level much higher than the noise generated by the propellers and that it would saturate the MEMS microphones of the array during a short interval. During explosions, the system would not be able to detect people’s cries for help. However, as people would continue to cry for help, it would be possible to detect them between explosions.

Another idea obtained from the joint work of the Array Processing Group and the Robotics Institute was the use of the reflections associated to the walls of the enclosed environments in order to obtain a 3D position estimation of people. Some tests have been carried out in order to locate the angles of arrival of different sound sources, including whistles and calls for help, in indoor environments. In the tests carried out in this paper, the acoustic array was not integrated on the drone, due to its high dimensions. Equivalent scenarios were built in order to evaluate the acoustic localization algorithms in the presence of the noise generated by the drone propellers.

Section 2 introduces the description of the system used in this study, showing the different hardware platforms that compose the system, and the system features. Section 3 presents the results obtained on the tests that were carried out in the analysis, and the corresponding discussion of these results. Finally, Section 4 contains the conclusions that authors have drawn on the basis of the obtained results.

## 2. Materials and Methods

The work shown in this paper is focused on using an unmanned aerial vehicle (UAV), or drone, to locate people in enclosed environments with reduced visibility. The objective of this work is to analyze if an acoustic array, placed on an operative UAV, can localize people cries, discriminating between the desired acoustic signals and the noise of the drone propellers.

### 2.1. Drone

**Figure 1:**
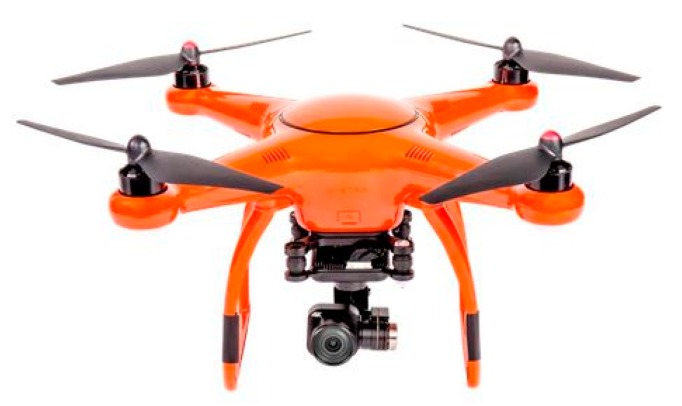
Autel X-star drone [37].

As shown in Figure 1, the drone that has been employed in this work is the Autel X-Star model. This drone is a quadrotor micro aerial vehicle. It has a 4K camera and a 3-axis gimbal that can take smooth Ultra HD video or 12MP photos. An intelligent flight control system and autopilot functions power the quadcopter. Its Dual GPS and GLONASS navigation System, the Starpoint Positioning System, and exclusive SecureFly technology keep the drone safe and stable, even in challenging situations.

### 2.2. Acoustic System Based on MEMS Array and a myRIO Platform

**Figure 2:**
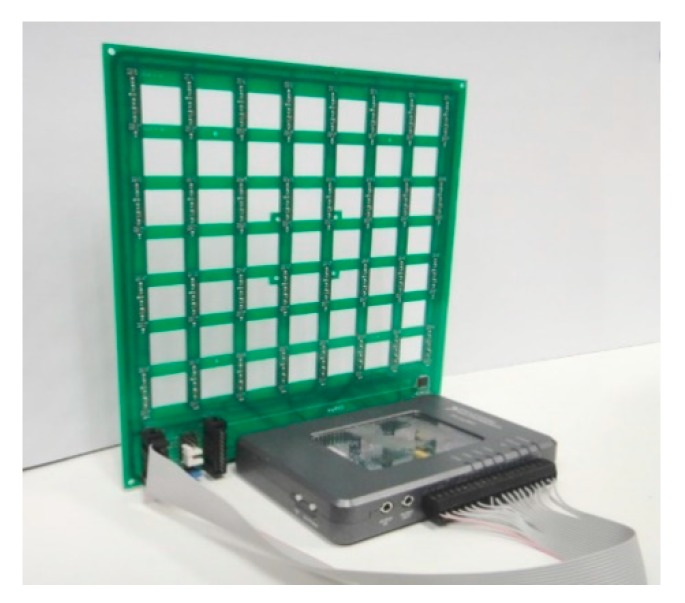
Array module with myRIO and MEMS array board.

The acoustic images acquisition system used in this paper is based on a Uniform Planar Array (UPA) of MEMS microphones [29]. This array, entirely developed by the authors from de University of Valladolid, is a square array of 64 (8 × 8) MEMS microphones that are uniformly spaced, every 2.125 cm, in a rectangular printed circuit board (PCB), as shown in Figure 2.

This array was designed to work in an acoustic frequency range between 4 and 16 kHz. Therefore, the 2.125 cm spacing corresponds to λ/2 for the 8 kHz central frequency. This spacing allows a good resolution for low frequencies, and also avoids grating lobes for high frequencies in the angular exploration zone of interest.

For the implementation of this array, MP34DT01 digital MEMS microphones of STMicroelectronics—with PDM interface—were chosen, with the following features: low-power, omnidirectional response, 63 dB SNR, high sensitivity (−26 dBFS) and a nearly flat frequency response (±6 dB in the range of 20 Hz to 20 kHz).

**Figure 3:**
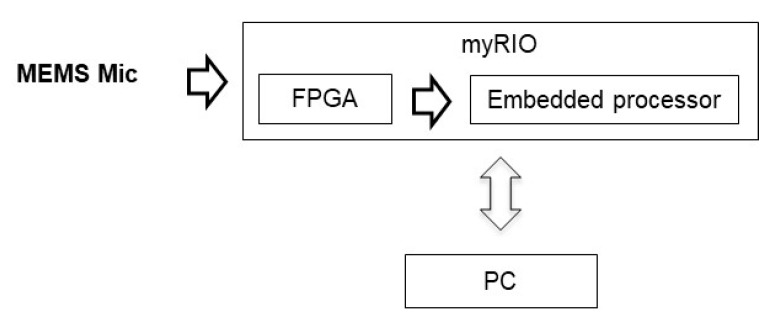
Acoustic system hardware setup.

A MyRIO platform [38] is the base unit for this system. This platform belongs to the reconfigurable input-output (RIO) family of devices from National Instruments, which is oriented to sensors with nonstandard acquisition procedures. The embedded processor included in myRIO can run all software algorithms to generate acoustic images, so it can be used as a standalone array module formed by a myRIO connected to a MEMS array board, as shown in Figure 1. In this work, the myRIO platform works as a standalone system, controlled from a PC connected using a Wi-Fi interface. Figure 3 shows the acoustic system hardware setup.

**Figure 4:**
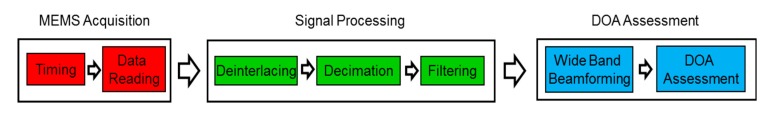
Processing algorithms.

The algorithms implemented in the system [29], shown in Figure 4, can be divided into three blocks: MEMS acquisition, signal processing, and direction of arrival (DOA) assessment (using wideband beamforming). The programming language used is LabVIEW, along with its Real Time and FPGA, modules, which allows developing applications on different hardware platforms such as those used in the system: FPGA, Embedded Processor (EP) and PC.

- In the acquisition block, each MEMS microphone performs signal acquisition with a 1-bit sigma-delta AD converter working at high speed (sample frequency of 2 million Samples per second).
- In the signal processing block, several routines are implemented: first the data from MEMS microphone are deinterlaced and then they are decimated obtaining 64 independent signals with a sampled rate of 50 kHz (one from each MEMS of the array). Finally, each signal is filtered with a high-pass filter in order to eliminate the low-frequency noise from the drone propellers or other sources like environmental noise or fire that have a low-frequency spectrum.
- The DOA block estimates the angles of arrival of direct and reflected signals. First, an acoustic image is obtained representing the sound pressure level (SPL) received for a set of NxN arrival directions in azimuth and elevation. From this acoustic image the angular coordinates of the local maximums are extracted, which are the arrival angles of the direct and reflected signals. The acoustic image is obtained by a deterministic wideband beamforming algorithm [23,24,25]. This algorithm can be considered as a spatial filter to estimate the sound pressure level (SPL) for a given direction of arrival.

Although, in this first stage, the signals captured by the microphones are transmitted through the Wi-Fi interface to a PC where the DOA is assessed. This processing could be done on the ARM processor included in the myRIO platform for autonomous operation.

### 2.3. Acoustic Localization Using the Direction of Arrival in an Enclosed Environment

**Figure 5:**
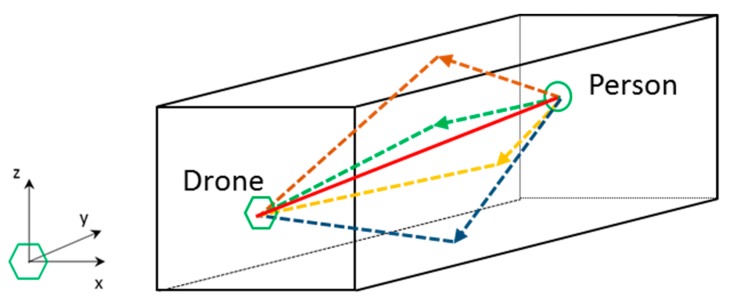
Direct (black) and reflected (colored) acoustic signals in an indoor space.

In an indoor space, typically reverberant, the acoustic distress signal issued by a person is propagated directly and through reflections. In a room/corridor, with a rectangular geometry, there will be typically 5 significant signals: the direct signal (red solid line) and 4 reflected signals/echoes (dashed lines) from each of the 4 reflection surfaces: floor (blue), ceiling (brown), left side wall (green) and right side wall (yellow); as it is shown in Figure 5.

Taking into account that the drone knows its position inside the indoor space, as well as geometry of the space, the position of the person to be located can be estimated. With this objective, the arrival angles (azimuth and elevation) of the direct signal and the reflected signals received by the acoustic array boarded on the drone are used to estimate the position of the person. The hypothesis of specular reflection on surfaces is assumed.

With the angle of arrival information (azimuth and elevation) of the direct signal, the drone can fly towards the person for a visual identification. In addition, if the angles of arrival of the four reflected signals are used, the 3D position of the person can be estimated by triangulation. By means of this technique, it is not necessary to estimate the range of the acoustic received signals, typical of an active SONAR system, based on the pulse-echo concept.

**Figure 6:**
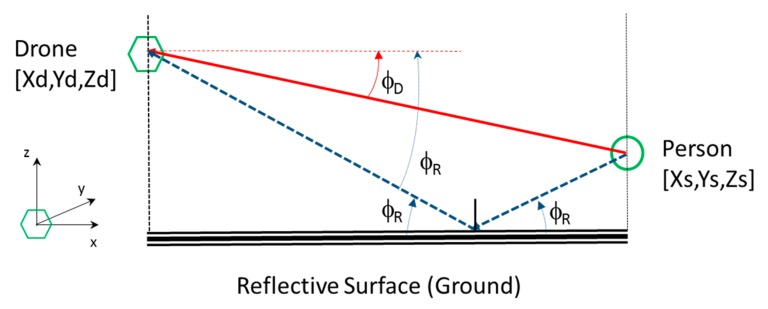
Angles of arrival in elevation.

As an example, Figure 6 shows the elevation angles of the direct (red solid line) and reflected (blue dashed line) signals in the YZ plane that contains the drone and the sound source, assuming that the floor of the room is the reflective surface.

The acoustic array estimates the angles of arrival, in elevation, of the direct signal ϕ_D_, and the reflected signal ϕ_R_, knowing the height Z_d_ of the drone with respect to the reflection surface. An equivalent estimator could also be obtained by using the ceiling as the reflection surface. Analogously, the arrival angles in azimuth of the direct signal and the signals reflected in each of the sidewalls of the room could be analyzed in the XY plane.

Merging the estimations of the arrival angles (azimuth and elevation) in the two orthogonal planes of analysis, the absolute position of the acoustic source [X_s_, Y_s_, Z_s_] is obtained, as a function of the absolute position of the drone [X_d_, Y_d_, Z_d_], by simple trigonometric relationships. As an example, based on Figure 6, Equations (1) and (2) show the expressions that can be used to obtained X_s_ and Z_s_ coordinates of the acoustic source position, knowing the drone position.

(1) $$X_{s}=X_{d}+2Z_{d}\frac{\cos\Phi_{R}\cdot \cos\Phi_{D}}{\sin\left(\Phi_{R}+\Phi_{D}\right)}$$

(2) $${\;Z}_{s}=Z_{d}\frac{\sin\left(\Phi_{R}-\Phi_{D}\right)}{\sin\left(\Phi_{R}+\Phi_{D}\right)}\;$$

In each analysis plane, two independent estimators can be obtained, related to the two reflection surfaces: the floor and the ceiling in elevation, and the left sidewall and the right sidewall in azimuth.

The equations proposed in this section are suitable for simple environments such as those proposed in the next section. In real scenarios, rooms contain more than 6 reflective surfaces, and these are not perpendicular to each other. As a result, the number of reflections increases considerably, making it difficult to estimate the direct signal direction. Other multi-source estimation algorithms such as those based on TDoAS [39,40] will be preferable in these cases.

## 3. Results

The objective of this publication is to determine the feasibility of using an array of MEMS microphones together with a 2D beamforming algorithm to determine the position of a sound source in an enclosed space and in the presence of a drone in operation.

The 3D position of the received signal could be estimated using the direct and reflected signals, while knowing the geometry of the workspace and the position of the drone in it (provided by the SLAM system). This estimation can be done without using an active system that transmits a pulsed signal and calculates the distance of the received echo. In this way, a passive location system can be used.

In a real system, the 2D array should be shipped inside the drone, but in order to simplify and accelerate the experiment, the employed work procedure anchored the drone to a fixed position and placed the 2D array in its vicinity. A speaker, excited with a set of test signals, generates the acoustic source. This speaker is placed in a predefined spatial position, determining the arrival angles of the direct signals, as well as the signals reflected in the surfaces under analysis.

In order to analyze the problem in different situations, three scenarios with different configurations have been arranged:

- Setup 1: In this setup, the drone is anchored to a horizontal surface and the 2D array is placed at 100 cm. This distance is the same that have been used to measure the background noise levels with a sound level meter. This setup allows the characterization of the noise generated by the four propellers of the drone.
- Setup 2: In this case, the 2D array is placed at 20 cm from the drone, in order to be as close as possible to it. In this setup, the emitting source is placed at 45 cm, which is the minimum distance that the drone could approach the person, because of its physical dimensions. This second setup allows the estimation of the DOA of a nearby emission source.
- Setup 3: In this case, the emitting source has been placed at 160 cm from the 2D array. This setup also adds one horizontal surface and one vertical one, in order to reflect the emitted acoustic signal mention before. The distance between the array and the sound source has been defined on the basis of the physical dimensions of the test area and the size and position of the employed reflective surfaces. This last setup allows the estimation of the DOAs of the direct signal and the reflections, of this direct signal, on a horizontal plane and on a vertical plane.

The challenge of the tests is the acquisition of the DOA of some acoustic signals, by discriminating these acoustic signals from the noise generated by the drone. Therefore, in these tests, there are two different kinds of sound sources:

- The acoustic signals to be detected. Two different acoustic sources have been used in this work:
  - A sinusoidal pulsed signal.
  - A continuous voice signal from a radio newscaster.
- The background noise, which in this case is the noise generated by the drone, which is produced by its four propellers. For the tests carried out, three background noise levels have been identified taking into account the different situations of the drone:
  - Minimum: When the drone is on a stationary situation (hovering).
  - Medium: When the drone describes slow horizontal movements.
  - Maximum: When the drone describes fast movements or it is taking off, that is moving vertically.

The associated noise levels for these situations have been measured with a sound level meter, placed 100 cm away from the UAV, in order to not interfere with the noise measurements. Table 1 shows the range of variation of the sound pressure level for each defined background noise situation along with its typical value.

### 3.1. Frequency Analysis of the Noise Generated by the Drone Propellers

**Figure 7:**
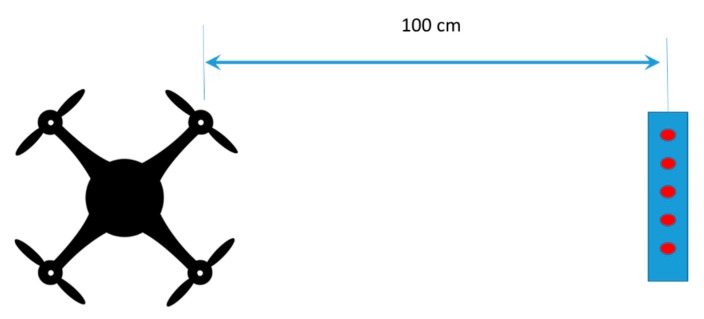
Elements distribution on Setup 1.

For this experiment, setup 1 has been used, as shown in Figure 7.

**Figure 8:**
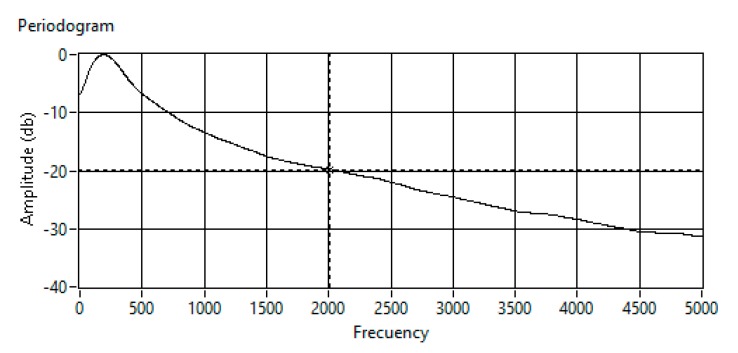
Averaged periodogram of the drone noise.

A 100-ms signal is acquired, obtaining an averaged periodogram in intervals of 5 ms for each of the 64 MEMS microphones. Finally, the averaged periodogram for the 64 channels is displayed. An example of this averaged periodogram is shown in Figure 8.

It is observed that the bandwidth of the noise generated by the drone is mostly comprised from 0 to 2 kHz. This information is essential to exclude these frequency bands in the beamforming algorithm. A cutoff frequency of 20 kHz assures a 20-dB noise reduction in the detection frequency band. Therefore, the frequency band from 2 kHz will be used to identify the angles of arrival of the distress signals of interest and frequencies bellow 2 kHz are filtered in the signal processing block of the processing algorithm.

In addition, discarding these frequencies allows having a better spatial resolution to identify the position of the acoustic source. Therefore, the spectral band of 2 kHz to 10 kHz will be used to perform the spatial processing on the distress signals to detect them.

### 3.2. Estimation of the Angle of Arrival for a Tone Pulsed with the Drone’s Noise

**Figure 9:**
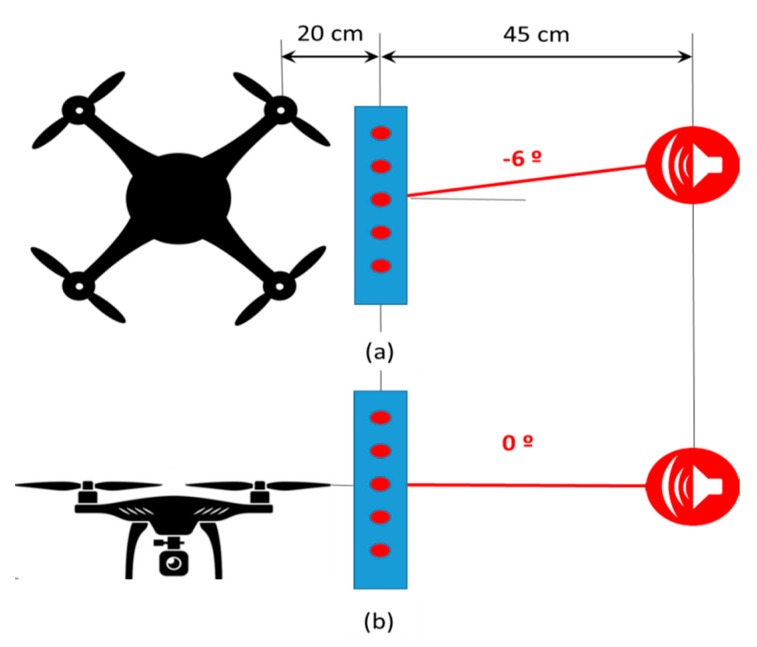
Elements distribution on Setup 2: (**a**) elevation view, (**b**) profile view.

For this experiment, setup 2 has been used, as shown in Figure 9. In these tests, the acoustic signal arrived the array from the (−6°, 0°) direction, in azimuth and elevation coordinates.

The objective of this experiment is twofold: (i) determining if the system can obtain the angle of arrival of the signal of interest, and (ii) evaluating if the noise of the four propellers of the drone degrade the results by MEMS saturation.

**Figure 10:**
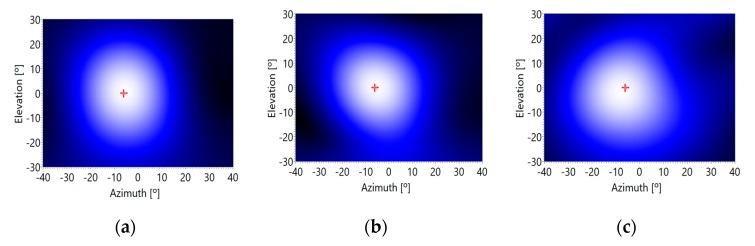
Acoustic image versus background noise level: (**a**) minimum, (**b**) medium and (**c**) maximum.

For this experiment, a 5-kHz sinusoidal pulse of 5 ms width has been generated, and 200 captures have been done for the three background noise levels: minimum, medium and maximum. First, a high-pass filter with a cut-off frequency of 2 kHz processed the received signals and then the acoustic images were obtained using a deterministic beamforming algorithm based on a 64-point FFT. Figure 10 presents the acoustic images corresponding to this sinusoidal pulse. In this figure, a red cross represents the real position of the acoustic signal.

The energy of the 5 ms sinusoidal pulse has been adjusted in order to have a typical SNR of 15 dB, 0 dB and −15 dB, for the three defined noise levels generated by the four propellers of the UAV, at the output of the high-pass filter. The tests have been carried out 200 times, and the mean and the standard deviation of the direction of arrival (DOA) angles, in azimuth and in elevation, have been assessed. Table 2 shows the obtained results.

Data in Table 2 show how accurate the angle of arrival estimation is, for minimum and medium background noise levels, that is, when the drone is on a stationary situation or it is moving slowly and horizontally. A bias of 2° and also a deviation of up to 2° have been detected. However, the estimation undergoes a significant deviation for the maximum background noise level, that is, when the drone moves fast and/or it is moving vertically. It would be advisable to keep the drone steady, every certain time interval, to estimate the angle of arrival accurately. Nevertheless, the error obtained for a high noise level, still allows having a rough estimation of the spatial position of the acoustic signal.

### 3.3. Estimation of the Angle of Arrival for a Tone Pulsed with the Drone’s Noise and Two Reflective Surfaces

**Figure 11:**
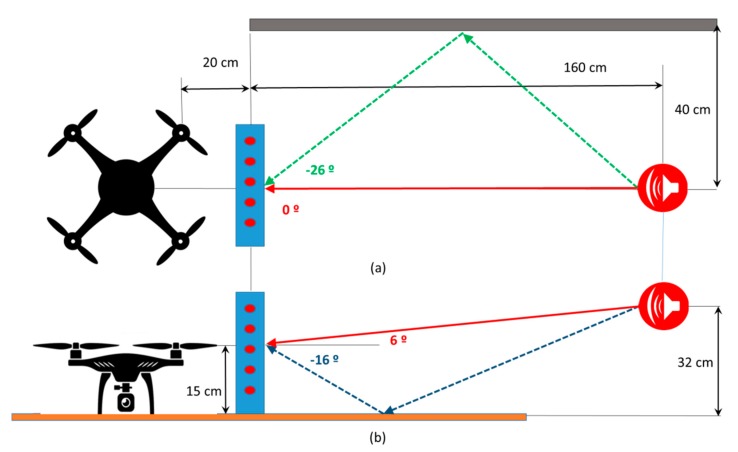
Elements distribution on Setup 3: (**a**) elevation view, (**b**) profile view.

For this experiment, setup 3 has been used, as shown in Figure 11. In this case, the direct sound reaches the array from the (0°, 6°) direction. As this setup also adds two reflective surfaces, located on a horizontal plane and on a vertical one, the emitted sound is reflected on them, generating two reflective signals or echoes, coming from the directions (0°, −16°) and (−26°, 6°), respectively.

The objective of this experiment is to determine if the system can get, together with the direct angle, the two reflective angles produced by the two interposed surfaces. As can be seen in Figure 11, the vertical surface should generate a reflected signal with an angle of −26° in azimuth, maintaining the angle of the speaker on 6° in elevation. On the other hand, the horizontal surface should generate a reflected signal with an angle of −16° in elevation, maintaining the angle of the speaker on 0° in azimuth.

**Figure 12:**
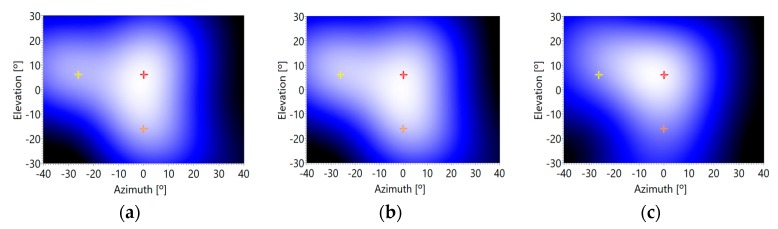
Acoustic image versus background noise levels: (**a**) minimum, (**b**) medium and (**c**) maximum.

In addition, for this experiment, a 5 ms sinusoidal pulse has been generated, making multiple captures for the three background noise levels: minimum, medium, and maximum. Figure 12 presents the acoustic images corresponding to the sinusoidal pulse.

It can be observed how the estimations of the direct and reflected angles of arrival are quite accurate for low and medium noise levels; and it suffers a deviation for high noise levels. The numeric data of the deviations can be observed in Table 3. In this case, the methodology also shows a bias of 3° and also a deviation of up to 3°. Also, in this case, the methodology works accurately with low and medium background noise conditions, but its accuracy falls if the background noise level increases.

### 3.4. Estimation of the Angle of Arrival for a Distress Signal with the Drone’s Noise and Two Reflective Surfaces

For this experiment, setup 3 has been used again, but in these tests the loudspeaker, which emulates the position of the sound source to be located, reproduces the signal of a radio newscaster with duration of 50 ms and a bandwidth of 10 kHz, which is processed in 2 ms segments. Multiple captures have been made for the three background noise levels: minimum, medium and maximum.

The objective of this experiment is to determine the capacity of the system to estimate the direct and reflected angles of arrival of a broadband sound source. In this case, an additional difficulty appears, because the voice signal has variable energy and spectral content over time, which requires averaging the acoustic images both in the frequency domain and in the time domain in order to obtain a reliable estimator.

**Figure 13:**
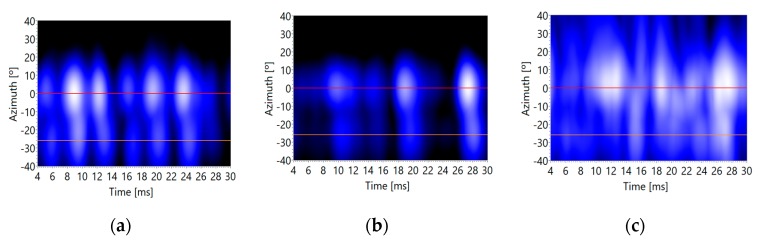
Acoustic image section (azimuth/time) versus background noise levels: (**a**) minimum, (**b**) medium and (**c**) maximum.

**Figure 14:**
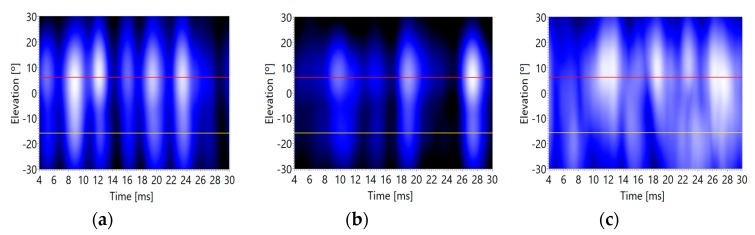
Acoustic image section (elevation/time) versus background noise levels: (**a**) minimum, (**b**) medium and (**c**) maximum.

Figure 13 and Figure 14 show different sections of the acoustic image for each segment of 2ms, where the acoustic images obtained have been averaged for a frequency range between 2 kHz and 10 kHz. In these figures, it can be observed that the energy contained in each segment of 2 ms varies over time, but it always shows maximum values, when they appear, around the positions of the direct and reflected signals. These signal positions are marked these figures with colored lines: red (direct signal), orange (vertical surface echo) and yellow (horizontal surface echo).

**Figure 15:**
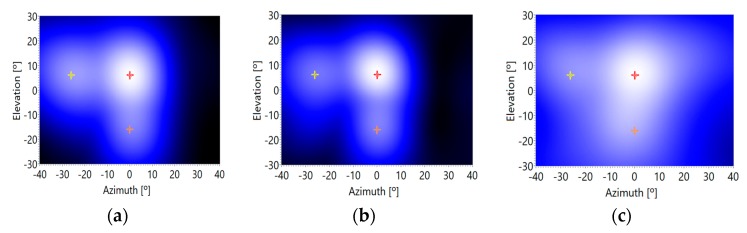
Time Averaged Acoustic image versus background noise levels: (**a**) minimum, (**b**) medium and (**c**) maximum.

Finally, the acoustic images for each time segment are averaged, obtaining a reliable image, where the angles of arrival of the direct signal and reflected ones can be estimated quite well, as shown in Figure 15, but with lower accuracy than in the previous tests, in the case of the reflective signals. As it can be observed in Figure 15, the voice signal and the reflected ones can be detected in the acoustic image around the corresponding locations: (0°, 6°) for the voice signal, (−26°, 6°) for the vertical plane reflective signal, and (0°, −16°) for the horizontal plane reflective signal, as indicated in Figure 11.

As well as in the previous tests, the detection of the DOA of the desired acoustic signals (direct and reflected ones) is accurate with low and medium background noise levels. Figure 15a,b show clear maxima at the positions of these desired signals. On the contrary, when the background noise is high, this methodology is not so accurate. In this case, desired signals are not so distinguishable from the background noise, as can be observed in Figure 15c.

It seems that if working with voice signals, the best behavior of the tests is, again, those associated to moderate noise environments. The numeric data of the deviations obtained in these tests can be observed in Table 4. The methodology shows a bias of 4° and a deviation of up to 12°. These data show that the accuracy of the methodology using voice signals is moderate.

### 3.5. Discussion

In summary, it can be observed that the use of an array of MEMS sensors together with beamforming techniques allows estimating the angular position of a distress signal as well as the reflections that this signal suffers in two known reflection planes, which emulate the walls, floors and ceilings of an enclosure. With the knowledge of these detected angles and the drone position, it is easy to obtain the position of the acoustic source, that is, of the person crying for help.

The drone’s propellers generate noise in the low frequency bands, which can be eliminated by filtering the signals in the temporal and spatial domain. In this way, a robust estimator of the angular position of a distress signal is obtained. It can be used on a people search system can use this estimator, that is based on a MEMS array boarded on a drone.

However, if the noise of the propellers is very strong, the performance of the system degrades significantly, so it is necessary to take at least two measures:

1. Acoustically isolation of the MEMS array from the noise generated by the propellers that will typically come from the back of the array.
2. Sequentially, keeping the drone in stationary flight, in order to minimize the noise generated, and thus to allow an accurate estimation of the angular coordinates of the direct and reflected distress signals.

## 4. Conclusions

In view of the results obtained in the work presented in this paper, it is feasible to estimate the angles of arrival of direct and reflected voice signals in an indoor space, using an array of MEMS microphones shipped on a drone. Due to the characteristics of the system, this voice signals have superimposed the noise generated by the propellers of the drone, but this does not mean a problem on the angles of arrival estimation. Given that the noise of the drone is essentially concentrated at low frequencies, this fact has enabled the system to discriminate between this noise and the voice signals.

For the proper system performance, it is important to ensure that the noise level does not saturate the MEMS sensors. Experiments with low and medium noise levels have yielded good results. It seems reasonable to isolate mechanically and acoustically the MEMS array from the structure of the drone while from time to time leave the drone hovering to better estimate the position of the acoustic signals received.

This methodology can work with any type of drone, adjusting the frequency parameters to the characteristics of the drone, based on the used motors, propellers, and rotation speeds. As a future work, it would be interesting to validate it with other drones in the market.

In other future works, an array of 10 × 10 cm will be integrated into a new drone, using a 2D gimbal to stabilize its position. The drone will house all the systems described in this paper, in order to analyze its performance in real situations. In this new integrated system, the information about the position of the acoustic sources will be sent to the drone controller in order to send it automatically to the area where the presence of people crying are detected.

This work has been the result of research collaboration between Professor Sebastian Sherer’s team, from the Robotics Institute of the Carnegie Mellon University, and the Array Processing research group of the University of Valladolid, led by Professor Alberto Izquierdo.

## Figures and Tables

**Table 1:** Noise levels generated by the propellers of the UAV for the defined background noise situations.

Background Noise Levels	Range (dB)	Typical Lp (dB)
Minimum	70–80	75
Medium	80–100	90
Maximum	100–120	105

**Table 2:** Accuracy data obtained for setup 2 tests.

Background Noise Levels	SNR (dB)	Azimuth (Mean ± Standard) Deviation	Elevation (Mean ± Standard) Deviation
Minimum	15	−6° ± 0.1°	0° ± 0.1°
Medium	0	−6° ± 0.2°	0° ± 0.1°
Maximum	−15	−8° ± 1.9°	−2° ± 1.2°

**Table 3:** Accuracy data obtained for setup 3 tests, using a pulsed acoustic signal.

Background Noise Levels	SNR [dB]	Azimuth (Mean ± Standard Deviation)			Elevation (Mean ± Standard Deviation)		
		Acoustic Signal	Vertical Plane	Horizontal Plane	Acoustic Signal	Vertical Plane	Horizontal Plane
Minimum	15	0° ± 0.1°	−26° ± 0.1°	0° ± 0.1°	6° ± 0.1°	6° ± 0.2°	−16° ± 0.2°
Medium	0	0° ± 0.2°	−26° ± 0.2°	0° ± 0.2°	6° ± 0.2°	6° ± 0.3°	−16° ± 0.4°
Maximum	−15	−2° ± 1.3°	−24° ± 1.8°	3° ± 2.1°	7° ± 1.8°	8° ± 2.8°	−13° ± 3.0°

**Table 4:** Accuracy data obtained for setup 3 tests, using a voice signal.

Background Noise Levels	SNR [dB]	Azimuth (Mean ± Standard Deviation)			Elevation (Mean ± Standard Deviation)		
		Voice Signal	Vertical Plane	Horizontal Plane	Voice Signal	Vertical Plane	Horizontal Plane
Minimum	15	0° ± 0.3°	−26° ± 0.4°	0° ± 0.5°	6° ± 0.5°	6° ± 0.7°	−16° ± 0.8°
Medium	0	0° ± 0.6°	−26° ± 0.8°	0° ± 1.0°	6° ± 0.8°	5° ± 1.1°	−16° ± 1.3°
Maximum	−15	1° ± 4.8°	−24° ± 7.0°	−4° ± 8.1°	7° ± 7.0°	5° ± 11.0°	−13° ± 12.1°
